# Time‐of‐day control of mitochondria regulates NLRP3 inflammasome activation in macrophages

**DOI:** 10.1096/fj.202400508RR

**Published:** 2024-12-17

**Authors:** James R. O'Siorain, Shannon L. Cox, Cloé Payet, Frances K. Nally, Yan He, Tabea T. Drewinksi, Oran D. Kennedy, Jennifer K. Dowling, Mark Mellett, James O. Early, Annie M. Curtis

**Affiliations:** ^1^ Curtis Clock Laboratory, School of Pharmacy and Biomolecular Sciences (PBS) Royal College of Surgeons in Ireland (RCSI) Dublin Ireland; ^2^ Institute of Functional Nano & Soft Materials (FUNSOM), Jiangsu Key Laboratory for Carbon‐Based Functional Materials and Devices Soochow University Suzhou China; ^3^ Tissue Engineering Research Group (TERG) Royal College of Surgeons in Ireland (RCSI) Dublin Ireland; ^4^ Brain Inflammation Group Ireland, School of Pharmacy and Biomolecular Sciences (PBS) Royal College of Surgeons in Ireland (RCSI) Dublin Ireland; ^5^ FutureNeuro SFI Research Centre Royal College of Surgeons in Ireland (RCSI) Dublin Ireland; ^6^ Department of Dermatology University Hospital Zürich (USZ), University of Zürich (UZH) Zürich Switzerland; ^7^ Faculty of Medicine University of Zürich (UZH) Zürich Switzerland

**Keywords:** circadian rhythms, macrophages, mitochondria, NLRP3 inflammasome, pyroptosis

## Abstract

Macrophages are innate immune cells that orchestrate the process of inflammation, which varies across time of day. This ensures appropriate biological timing of the immune response with the external environment. The NLRP3 inflammasome mediates IL‐1‐family cytokine release via pyroptosis. Mitochondria play a multifaceted role regulating NLRP3 inflammasome activity. Mitochondria exhibit distinct metabolic changes across time of day, which are influenced by clock genes. However, whether the macrophage clock regulates the NLRP3 inflammasome via mitochondrial control remains unclear. We find heightened mitochondrial membrane potential (Δψm) and enhanced NLRP3 inflammasome activation from peritoneal exudate cells (PECs) isolated at circadian time (CT) 12 compared to CT 0. In vitro time‐of‐day synchronization of bone‐marrow derived macrophages (BMDMs) induced time‐dependent differences in NLRP3 inflammasome activation. Myeloid‐specific *Bmal1*‐deletion enhanced NLRP3 inflammasome activity in PECs at CT0 and in unsynchronized BMDMs compared to controls. Pharmacologically disrupting Δψm in synchronized cells reduced NLRP3 inflammasome activation to comparable levels, and the same occurred with *Bmal1*‐deletion. These results further demonstrate circadian clock timing of the NLRP3 inflammasome, which is dependent on mitochondrial function and driven through the circadian gene *Bmal1*.

## Graphical abstract

The circadian clock, orchestrated by BMAL1, regulates the expression of *Nlrp3* and *Il1b* in response to lipopolysaccharide (LPS), causing time‐of‐day production of pro‐IL‐1β. Mitochondria exhibit time‐of‐day changes in mitochondrial membrane potential (ΔΨm). Caspase‐1 (CASP1) and gasdermin‐D (GSDMD) activation in response to NLRP3 inflammasome activation is dependent on ΔΨm. Both time‐of‐day transcriptional control and ΔΨm contribute to the circadian regulation of the NLRP3 inflammasome.

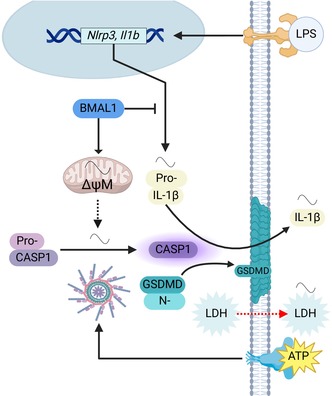

**Abbreviations**
ATP: adenosine triphosphate
BMAL1: brain and muscle arnt‐like protein 1
BMDMs: bone marrow‐derived macrophages
CASP1: caspase‐1
CT: circadian time
FCCP: carbonyl cyanide 4‐(trifluoromethoxy)phenylhydrazone
GSDMD: gasdermin‐D
i.p.: intraperitoneal
IL‐1β: interleukin‐1β
LDH: lactate dehydrogenase
LPS: lipopolysaccharide
NLRP3: NLR family pyrin domain containing 3
PECs: peritoneal exudate cells
PRR: pattern recognition receptor
PS: post‐synchronization
redox: reduction–oxidation
ROS: reactive oxygen species
ZT: zeitgeber time
Δψm: mitochondrial membrane potential

## 1 INTRODUCTION

Life on Earth has evolved to anticipate the recurring environmental changes of light and dark by endogenously tracking time via the circadian molecular clock. Timekeeping extends to the cellular level with circadian genes, such as *Bmal1*, comprising molecular clocks.^1^ Circadian molecular clocks utilize a series of feedback loops in gene transcription and protein expression. In addition, reduction–oxidation (redox) reactions and metabolic flux drive circadian rhythmicity.^2^ Circadian clock disruption, which can occur through jet lag, shift work and light at night, is a risk factor for chronic inflammatory diseases.^3^ Macrophages are innate immune cells that play a dominant role in the orchestration of the inflammatory response through the production of cytokines. Furthermore, many chronic human diseases driven by macrophage‐derived inflammatory cytokines exhibit a circadian rhythm in symptom severity, for example, asthma,^4^ rheumatoid arthritis,^5^, ^6^ and psoriasis.^7^ Despite these observations, our molecular understanding of how macrophages contribute to chronic inflammatory disease pathology remains incomplete.

The NLRP3 inflammasome is central to the macrophage inflammatory response by processing and releasing inflammatory cytokines, such as interleukin‐1β (IL‐1β), to amplify inflammation.^8^ In murine macrophages, a 2‐step mechanism of PRR‐mediated “priming” and “activation” induces NLRP3 inflammasome activity.^9^ Priming drives the protein expression of inflammasome components such as NLRP3 and pro‐IL‐1β, whereas the assembly of the ASC‐speck and activation of caspase‐1 (CASP1) marks inflammasome activation. CASP1 liberates gasdermin‐D (GSDMD) auto‐inhibition and facilitates GSDMD N‐terminal (NT) pore formation in the plasma membrane.^10^, ^11^, ^12^, ^13^ GSDMD pores disrupt the osmotic balance causing cells to swell, plasma membranes to rupture, and facilitating the release of pro‐inflammatory cytokines and alarmins in a programmed cell death pathway, termed pyroptosis.^14^, ^15^ IL‐1β causes the inflammatory activation of IL‐1‐responsive cells with a further role in circadian inflammation. Indeed, we have recently demonstrated that fibroblasts respond to IL‐1β by regulating the production of CXCL5 according to time of day through BMAL1's inhibition of NF‐κB activation.^16^

Mitochondria play a complex role in the regulation of the NLRP3 inflammasome,^17^, ^18^ with alarmins, such as mitochondrial DNA (mtDNA) and reactive oxygen species (ROS), shown to enhance NLRP3 inflammasome activity.^14^, ^19^, ^20^, ^21^, ^22^ On the other hand, mitochondrial‐derived metabolites, such as fumarate and itaconate, limit NLRP3 inflammasome activity.^23^, ^24^, ^25^ Mitochondrial cardiolipin is critical to activation of the NLRP3 inflammasome as cardiolipin allows NLRP3 to co‐localize to mitochondria.^26^, ^27^ Recently, GSDMD mitochondrial pore formation was found to precede plasma membrane rupture, and this was dependent on cellular redox balance.^28^, ^29^ Furthermore, disruption of the mitochondrial electron transport chain (ETC) ablates NLRP3 inflammasome activity in macrophages, although the precise mechanism of how the ETC regulates the NLRP3 inflammasome remains elusive.^30^

The macrophage molecular clock is emerging as an important regulator of the NLRP3 inflammasome. Deletion of core circadian genes such as *Bmal1*, along with *Nr1d1*, which codes for Rev‐Erbα, drives enhanced NLRP3 inflammasome activation via loss of transcriptional regulation of *Nlrp3* and *Il1b*.^31^, ^32^, ^33^ Furthermore, there is growing evidence that the molecular clock is also important for mitochondrial function in myeloid cells.^34^, ^35^, ^36^, ^37^ Indeed, mitochondrial dynamics exhibit a circadian rhythm,^34^, ^35^, ^38^ and significant differences in myeloid cell mitochondrial metabolism exist with the deletion of *Bmal1*.^36^, ^37^ To date, the impact of circadian rhythms in mitochondria on regulating the NLRP3 inflammasome in macrophages remains to be investigated.

Herein, we show time‐of‐day control of the NLRP3 inflammasome, which is dependent on mitochondrial membrane potential (Δψm). We show that Δψm varies between peritoneal macrophages harvested at CT0 and CT12, with greater Δψm and NLRP3 inflammasome activation at CT12. We show that in vitro synchronization of the macrophage molecular clock induces time‐of‐day dependent differences in the NLRP3 inflammasome, with priming and activation enhanced at 28 h post‐synchronization, corresponding to CT12. We show that pharmacological inhibition of Δψm with FCCP, ablates time‐of‐day dependent NLRP3 inflammasome activation of CASP1 and GSDMD, leading to a significant reduction of IL‐1β release and cell death. We further demonstrate that deletion of *Bmal1*‐enhances NLRP3 inflammasome activity, which is also ablated with disruption of Δψm. This study highlights the importance of the molecular clock in regulating the NLRP3 inflammasome in macrophages and provides further insight as to how this occurs. Circadian disruption contributes to the break in endosymbiosis between mitochondria and the cell, and this may underlie inflammatory disease.^39^ This data provides reasoning for how circadian rhythms in chronic inflammatory disease occur, via time‐of‐day regulation of mitochondria and the NLRP3 inflammasome. Moreover, these findings support the potential therapeutic avenues for the timed administration of inflammasome‐targeting drugs in the treatment of chronic inflammatory disease.

## 2 MATERIALS AND METHODS

### 2.1 Animals

Mice containing a lysozyme M activated CRE recombinase (Lyz2Cre, Jackson Labs #004781) were crossed with mice with LoxP sites flanking both sides of exon 4 of *Bmal1* (Bmal1LoxP/LoxP, gifted from the lab of Dr. Christopher A. Bradfield) to generate mice with the Bmal1 gene excised specifically in the myeloid lineage (Bmal1LoxP/LoxPLyz2Cre), that is, *Bmal1* ^*−/−*^. Offspring were genotyped to confirm the presence of LoxP sites and Cre recombinase. *Bmal1* ^*−/−*^ mice were compared with control Lyz2Cre (*Bmal1* ^*+/+*^) mice. C57BL/6 wild‐type mice were bred in‐house from established colonies. Male mice were used for all experiments. All mice were housed in SPF conditions in the Comparative Medicine Unit, Trinity College Dublin. All mice were maintained in line with the Irish Health Products Regulatory Authority and European Union regulations. Experiments were carried out under HPRA license and with ethical approval from Trinity College Dublin bioethics committee and Royal College of Surgeons in Ireland ethics committee.

### 2.2 Isolation of bone marrow‐derived macrophages

The hind legs of mice were placed within sterile 35/10 mm petri dishes. To expose the tibia and fibula, scissors were used to sever below the hip and above the ankle. The knee joint and muscle tissue were cut away to separate the tibia and femur. Bone cavities were then flushed with PBS using a 25 g 1 mL syringe. Bone marrow cells suspended within the flow‐through were collected in a 10 mL falcon tube and dripped through a cell strainer. These falcon tubes were then centrifuged at 1500 rpm for 5 min. Supernatants were discarded and the pellets re‐suspended in 2 mL of red cell lysis buffer for 2–3 min and then made up to 30 mL with DMEM. Falcon tubes were centrifuged at 1500 rpm for 5 min; cell supernatants were removed, and pellets re‐suspended in 30 mL DMEM consisting of 10% FCS, 20% L929, and 1% P/S. These supernatants were equally distributed into 3 separate 10 mm petri dishes and were cultured for 6 days at 37°C. Every 3 days, 5 mL of fresh DMEM media (10% FCS, 15 ng/mL MCSF, and 1% P/S) was added.

### 2.3 Isolation of peritoneal exudate cells

For PECs isolation, a central abdominal incision was made, and the skin was pulled down over the hind legs. A 23G syringe was inserted into the peritoneal cavity, lavaged with 3 mL of sterile PBS, and collected with the same syringe. Following isolation of cells from the peritoneal lavage, the mixed population of cells were pelleted via centrifugation at 1500 rpm for 5 min. The pellet was re‐suspended in serum‐free DMEM media and the cells counted and plated at 1 × 10^6^/mL in a 12‐well plate. Cells were incubated for 30 min to ensure adherent populations have become bound to plate, which were considered as peritoneal macrophages. The media was then removed, and the plate was washed twice with warm DMEM to remove non‐adherent populations.

### 2.4 Lumicycler

PECs or BMDMs were seeded into 35 mm cell culture dishes at a minimum density of 1.5 × 10^6^ cells at 37°C in Lumicycler recording media made up of 500 mL ddH_2_O, 5 g DMEM Powder (Sigma, D2902), 1.75 g D‐glucose (Sigma, G7021), 5 mL HEPES (Gibco, 15630056), 5% FCS, 1% P/S, and 50 mg Beetle Luciferin (Promega, E1602). A syringe containing vacuum grease and 40 mm glass coverslips were irradiated with UV light for 30 min. Dishes were sealed using vacuum grease and coverslips before being placed into the 32‐channel Lumicycler by Actimetrics, which utilizes photomultiplier tubes. Phase and period were determined by the inbuilt Lumicycler Analysis software.

### 2.5 Western blotting

For protein quantification via western blot, supernatant samples were removed, and cells were lysed in Laemmli buffer. Supernatants were prepared using Strataclean Resin (Agilent, 400714). Protein samples were separated via SDS‐PAGE. The Bio‐Rad gel running system and wet transfer system were used to run and transfer proteins onto nitrocellulose membranes. Following protein transfer, membranes were blocked with 5% milk for 1 h before being incubated in primary antibodies for BMAL1 (14020S, CST), Pro‐IL‐1β (AF‐401‐NA, CST), β‐Actin (4967S, CST), GSDMD (G7422, Sigma Aldrich), CASP1 (22915‐1‐AP, Protein Tech), NLRP3 (D4D8T, CST) at 4°C overnight. Membranes were then washed 3 times with TBST followed by incubation of specified secondary antibodies for 2 h at room temperature. Visualization of proteins was carried out on Amersham 680 Imager (GE Healthcare). Densitometry was carried out using ImageLab software (V.6.1, BioRad).

### 2.6 qRT‐PCR

Total RNA was isolated using Purelink RNA mini‐isolation kit (12183025, Thermo Fisher Scientific) and reverse transcribed to cDNA using High‐Capacity cDNA Reverse Transcription Kit (4368813, Thermo Fisher Scientific) using the ProFlex™ PCR system. For qPCR, the following primers were used: *18 s* (Forward: 5′‐CCCTCTATGGGCTCGAATTT‐3′, Reverse: 5′‐GGATGTGAAGGATGGGAAGT‐3′), *Nlrp3* (Forward: 5′‐GACACGAGTCCTGGTGACTT‐3′, Reverse: 5′‐CGTCTGTTGGTGATTGGCTT‐3′), *Il1b* (Forward: 5′‐TGGCAACTGTTCCTG‐3′, Reverse: 5′‐GGAAGCAGCCCTTCATCTTT‐3′), *Casp1* (Forward: 5′‐CATGCCGTGGAGAGAAACAAG‐3′, Reverse: 5′‐AGCCCCTGACAGGATGTCTC‐3′), *Gsdmd* (Forward: 5′‐GGCTGCATCCTTGAGTGTCT‐3′, Reverse: 5′‐AGACGTGCTTCACCAA CTCC‐3′), *Bmal1* (Forward: 5′‐TGCAATGTCCAGGAAGTTAGAT‐3′, Reverse: 5′‐GTTTGCTTCTGTGTATGGGTTG‐3′), and *Cry1* (Forward: 5′‐CTCGGTAGAGGAAGTCGGGG‐3′, Reverse: 5′‐TCAAGCAAAAATCGCCACCT‐3′). RT‐qPCR reaction was run using Applied Biosystems 7900HT PCR system using the PowerUp™ SYBRR Green Master Mix (A25778, Thermo Fisher Scientific).

### 2.7 Flow cytometry

For assessment of cellular ROS and mitochondrial membrane potential (Δψm), PECs or BMDMs were incubated with Fc block (101301, BioLegend), Zombie Near IR live dead stain (423105, BioLegend), MitoSpy Red CMXRos (424801, BioLegend) or CellROX™ Deep Red reagent (C10422, Invitrogen) at room temperature for 20 min. Unstained cells and single‐stained cells for each fluorophore were used for compensation controls. Cells were then washed with flow buffer, centrifuged at 300 *g* for 5 min, and fixed using Cyto‐Fast™ Fix/Perm Buffer Set according to manufacturer's protocol. Cells were then washed with flow buffer, centrifuged at 300 *g* for 5 min, resuspended in 200 μL flow buffer and left at 4°C overnight. Peritoneal B cells were defined as B220^+^ (103211, BioLegend) and peritoneal macrophages as CD11b^+^ (101222, BioLegend). Data were obtained using FACS Canto II or LSR Fortessa (BD) and analyzed using FlowJo (version 8).

### 2.8 NLRP3 inflammasome assays

BMDMs were synchronized with the supplementation of 50% horse serum for 2 h. Following synchronization, BMDMs were returned to DMEM (10% FCS, 15 ng/mL MCSF, and 1% P/S). NLRP3 inflammasome activation was carried out with treatment of 100 ng/mL LPS for 3 h to transcriptionally prime inflammasome components. The NLRP3 inflammasome was then activated with 5 mM ATP (tlrl‐atp, Invivogen) for 1 h. For mitochondrial inhibition experiments, medium was removed following LPS stimulation and replaced with serum‐free DMEM and treated with 100 nM FCCP (SML2959, Sigma‐Aldrich) for 1 h, prior to NLRP3 activation with ATP. Cell death was assayed using the LDH CytoTox 96® Non‐Radioactive Cytotoxicity Assay (G1780, Promega). Protein release of IL‐1β (DY401, Biotechne), IL‐18 (Abcam, ab216165), and TNFα (DY410, Biotechne) was quantified with ELISA.

### 2.9 ATP measurement

For measurement of cellular ATP, the ATP/ADP assay kit (ab83355, Abcam) was performed per manufacturer instruction. In brief, cell lysates were harvested from a 96‐well plate at a density of 5 × 10^4^ with 100 μL Assay Buffer and homogenized by pipetting. Insoluble material was removed by centrifuging at 10 000 *g* at 4°C. Supernatants were collected in new Eppendorf tubes. 50 μL samples, followed by 50 μL of Reaction Mix were added to a microplate. This plate was incubated at 37°C for 30 min then read at 570 OD.

### 2.10 Statistics

Statistical analyses were conducted using GraphPad Prism. All data are representative of at least *N* = 3 biological replicates from independent experiments. Data are presented as mean ± SEM. Two‐way ANOVA with Tukey's multiple comparisons test for grouped data or the Kruskal–Wallis with Dunn's multiple comparison test for nonparametric analyses were performed. Significance is reported as *p < .05, **p < .01, ***p < .005, and ****p < .001. *N* = numbers refer to the number of biological replicates used to repeat an experiment.

## 3 RESULTS

### 3.1 Time‐of‐day coordinates mitochondrial function and NLRP3 inflammasome activity

To begin to examine the intersection between circadian regulation, mitochondria, and the NLRP3 inflammasome within macrophages, *mPer2* ^*Luc*^ mice expressing PER2::luciferase were entrained to opposing 12‐h light–dark cycles for 2 weeks prior to 24 h in constant darkness. Peritoneal exudate cells (PECs) were isolated at CT0 (subjective lights on; start of rest phase), or CT12 (subjective lights off; start of active phase) (Figure S1A). As expected, the circadian period of each group approximated 24 h, whereas circadian phase varied by 12 h between CT0 and CT12 harvests (Figure S1B). Following isolation at CT0 and CT12, peritoneal macrophages were assessed for mitochondrial membrane potential (Δψm). We observed significantly higher Δψm at CT12 compared to CT0 (Figure S1C). However, there was no significant difference in Δψm of peritoneal B cells isolated at CT0 and CT12 (Figure S1D). To determine transcriptional changes in PECs across time of day, we examined the gene expression omnibus dataset GSE25585^40^ (Figure S1E,F). We detected rhythmic expression of *Il1b*, however did not observe rhythmic expression in other inflammasome genes (Figure S1G). Next, we assessed NLRP3 inflammasome activation from PECs at CT0 and CT12 and found that IL‐1β and lactate dehydrogenase (LDH) release was increased with NLRP3 inflammasome activation at CT12 compared to CT0 (Figure S1H,I). However, there was no significant difference in TNFα release from LPS‐stimulated PECs at CT0 and CT12 (Figure S1J). Overall, we find time‐of‐day differences in Δψm, with heightened Δψm at CT12 compared to CT0 which correlates to elevated NLRP3 inflammasome activation and pyroptosis at this time.

### 3.2 NLRP3 inflammasome activation is time‐of‐day dependent

**FIGURE 1:**
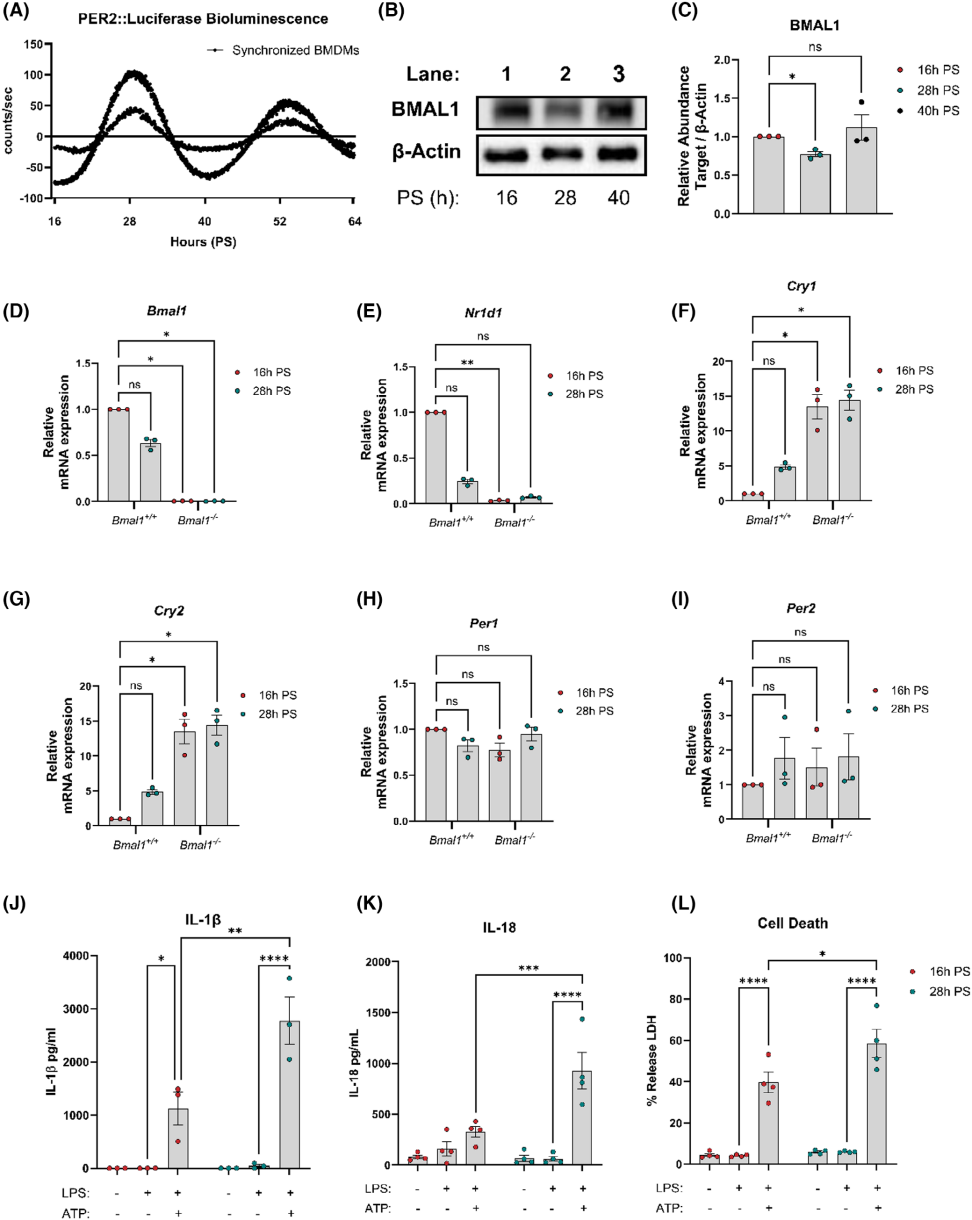
Clock timing impacts IL‐1 family cytokine release and cell death. (A) PER2::luciferase bioluminescent counts of synchronized BMDMs assessed for 48 h (*n* = 3). (B, C) BMAL1 protein expression from synchronized BMDMs (*n* = 3). (D–I) *Bmal1*, *Nr1d1, Cry1, Cry2, Per1, and Per2* mRNA expression of BMDMs 16 h and 28 h post‐synchronization (PS) (*n* = 3). (J) IL‐1β released from NLRP3 inflammasome activated BMDMs at 16 h PS versus 28 h PS (*n* = 3). (K) IL‐18 released from NLRP3 inflammasome activated BMDMs at 16 h PS versus 28 h PS (*n* = 4). (L) Cell death assayed via release of LDH from NLRP3 inflammasome activated BMDMs at 16 h PS versus 28 h PS (*n* = 4). Data are expressed as mean ± SEM. *N* numbers represent biological samples with technical duplicates. (A) Circadian parameters measured with MetaCycle JTK cycle analysis (period = 23.9 h, *p* < .001). (C–I) Statistical analyses were conducted using Kruskal–Wallis with Dunn's multiple comparisons test. (J–L) Statistical analyses were conducted using two‐factor analysis of variance (ANOVA) with Tukey's multiple comparisons test. *p < 0.05, **p < 0.01, ***p < 0.001 and ****p < 0.0001 and ns, non significant.

Following our observations of ex vivo timing of the NLRP3 inflammasome in PECs, we next investigated the mechanistic basis for this using in vitro models. First, we confirmed molecular clock synchronization of bone marrow‐derived macrophages (BMDMs) from *mPer2* ^*Luc*^ mice by monitoring PER2::luciferase activity (Figure 1A). We identified a robust circadian period of 23.9 h in synchronized BMDMs with peak PER2::luciferase activity at 28.6 h PS (Figure 1A). We additionally observed oscillations in BMAL1 protein expression, with reduced expression observed at 28 h post synchronization (PS) (Figure 1B,C). Furthermore, the gene expression of *Bmal1* (Figure 1D) and *Nr1d1* (Figure 1E) was trending lower at 28 h PS compared to 16 h PS in *Bmal1* ^*+/+*^ BMDMs. In *Bmal1* ^*−/−*^ BMDMs, *Bmal1* (Figure 1D) and *Nr1d1* (Figure 1E) expressions were significantly reduced but did not vary between 16 h and 28 h PS. The expression levels of *Cry1* (Figure 1F) and *Cry2* (Figure 1G) were trending higher at 28 h PS compared to 16 h PS in *Bmal1* ^*+/+*^ BMDMs and were significantly elevated in *Bmal1* ^*−/−*^ BMDMs. The expression of *Per1* (Figure 1H) and *Per2* (Figure 1I) did not vary between 16 h and 28 h PS in *Bmal1* ^*+/+*^ BMDMs or with *Bmal1* deletion. To assess time‐of‐day NLRP3 inflammasome activity, we triggered NLRP3 inflammasome activation at 16 h and 28 h PS. The release of IL‐1β, IL‐18, and LDH was greater with NLRP3 inflammasome activation at 28 h PS compared to activation at 16 h PS (Figure 1J–L). We conclude that NLRP3 inflammasome activation is dependent on time of day, such that molecular time in BMDMs controls the magnitude of IL‐1β, IL‐18 release and cell death in agreement with our observations ex vivo.

### 3.3 Priming of the NLRP3 inflammasome is controlled by molecular clock time

**FIGURE 2:**
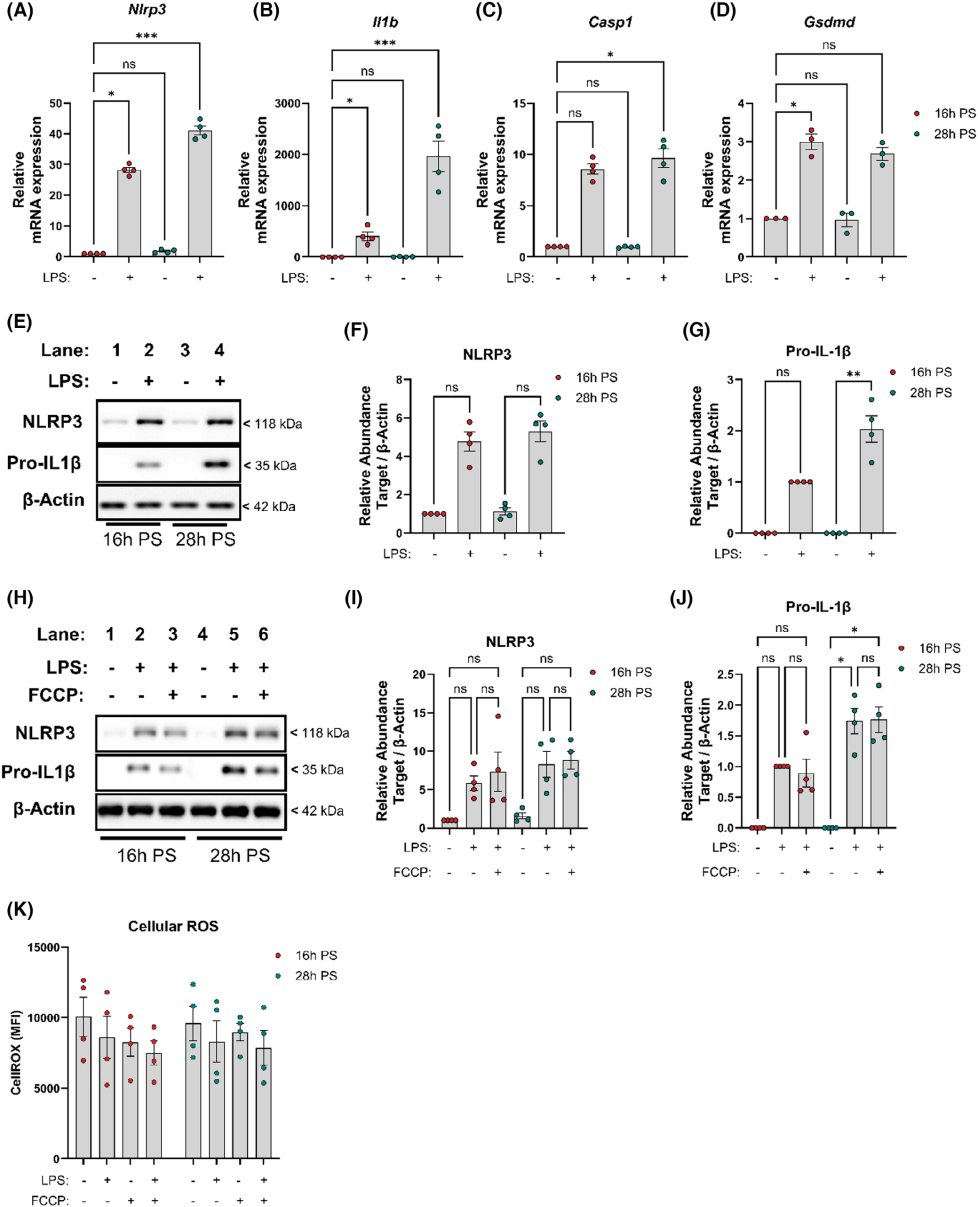
Time of day transcriptionally regulates levels of pro‐IL‐1β, independent of mitochondrial membrane potential. (A–D) *Nlrp3*, *Il1b, Casp1*, and *Gsdmd* mRNA expression of synchronized BMDMs treated with LPS (100 ng/mL, 3 h) 16 h PS and 28 h PS (*n* = 3). (E–G) NLRP3 and pro‐IL‐1β protein expression from LPS treated (100 ng/mL, 3 h) synchronized BMDMs at 16 h PS versus 28 h PS, with corresponding densitometry of relative quantity (RQ) normalized to β‐Actin (*n* = 3). (H, I) NLRP3 and pro‐IL‐1β protein expression from LPS (100 ng/mL, 3 h) and FCCP (100 nM, 1 h) treated synchronized BMDMs at 16 h PS versus 28 h PS, with corresponding densitometry of relative quantity (RQ) normalized to β‐Actin (*n* = 4). (K) Cellular reactive oxygen species (ROS) abundance measured via CellROX from synchronized BMDMs 16 h and 28 h PS treated with LPS (100 ng/mL, 3 h) followed by FCCP (100 nM, 1 h) (*n* = 4). Data are expressed as mean ± SEM. *N* numbers represent biological samples with minimum technical duplicates. (A–J) Statistical analyses were conducted using Kruskal–Wallis with Dunn's multiple comparisons test. (K) Statistical analyses were conducted using two‐factor analysis of variance (ANOVA) with Tukey's multiple comparisons test. *p < 0.05, **p < 0.01, ***p < 0.001 and ns, non significant.

To further understand how the circadian clock regulates the NLRP3 inflammasome, we next investigated the transcriptional priming of NLRP3 inflammasome components in BMDMs treated with lipopolysaccharide (LPS) at 16 h versus 28 h PS. *Nlrp3* mRNA expression (Figure 2A) and *Il1b* mRNA expression (Figure 2B) were markedly increased in LPS‐activated BMDMs at 28 h PS compared to 16 h PS. However, mRNA expression of inflammasome effectors *Casp1* (Figure 2C) or *Gsdmd* (Figure 2D) did not overtly change with time‐of‐day LPS treatments. The expression of NLRP3 was not different at 16 h versus 28 h PS; however, pro‐IL‐1β expression was significantly increased in response to LPS at 28 h (Figure 2E–G). To assess whether the expression of inflammasome components is affected by mitochondrial disruption, we used the protonophore FCCP, which uncouples ATP synthesis from mitochondrial electron transport by abolishing Δψm. However, there was no observed effect of mitochondrial uncoupling with FCCP after LPS priming in the expression of NLRP3 or pro‐IL‐1β at 16 h versus 28 h PS (Figure 2H–J). To establish whether differences in NLRP3 activation were due to changes in reactive oxygen species (ROS), we next examined the abundance of cellular ROS. We observed no significant differences in total cellular ROS abundance in response to LPS stimulation or with FCCP treatment at 16 h versus 28 h PS (Figure 2K). Inflammasome assembly is an ATP‐dependent process and as such, we also assessed whether differences in total ATP abundance exist between basal and LPS‐treated BMDMs at 16 h versus 28 h PS. However, we found no significant differences in ATP between 16 h and 28 h PS, with and without LPS (Figure S2). Cumulatively, these findings demonstrate that the molecular clock exerts transcriptional regulation of *Nlrp3* and *Il1b*, leading to time‐of‐day variation in pro‐IL‐1β expression in response to LPS.

### 3.4 NLRP3 inflammasome activation and pyroptosis are time‐of‐day controlled and dependent on mitochondrial membrane potential

**FIGURE 3:**
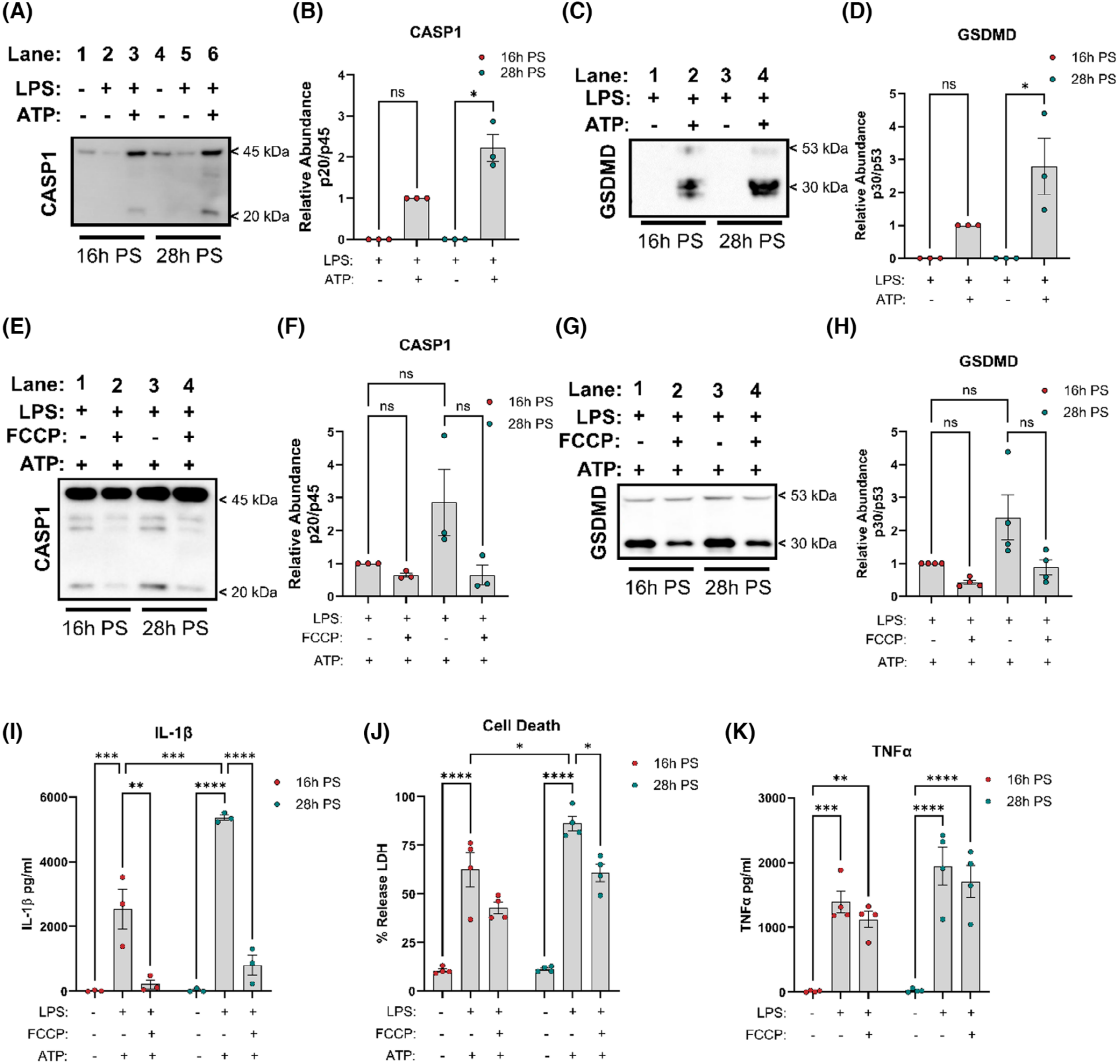
NLRP3 inflammasome activation is time‐of‐day regulated and dependent on mitochondrial membrane potential. (A, B) Caspase‐1 (CASP1) p45 and p20 supernatant expression following NLRP3 inflammasome activation at 16 h and 28 h PS (*n* = 3). (C, D) Gasdermin‐D (GSDMD) p53 and p30 supernatant expression following NLRP3 inflammasome activation at 16 h and 28 h PS (*n* = 3). (E, F) Caspase‐1 (CASP1) p45 and p20 supernatant expression following NLRP3 inflammasome activation with FCCP inhibition at 16 h and 28 h PS (*n* = 3). (G, H) Gasdermin‐D (GSDMD) p53 and p30 supernatant expression following NLRP3 inflammasome activation with FCCP inhibition at 16 h and 28 h PS (*n* = 3). (I) IL‐1β released from NLRP3 inflammasome activated synchronized BMDMs with FCCP inhibition at 16 h PS versus 28 h PS (*n* = 3). (J) Cell death assayed via release of LDH from NLRP3 inflammasome activated synchronized BMDMs with FCCP inhibition at 16 h PS versus 28 h PS (*n* = 4). (K) TNFα release from synchronized BMDMs 16 h and 28 h PS treated with LPS (100 ng/mL, 3 h) followed by FCCP (100 nM, 1 h) (*n* = 4). Data are expressed as mean ± SEM. *N* numbers represent biological samples with minimum technical duplicates. (A–H) Statistical analyses were conducted using Kruskal–Wallis with Dunn's multiple comparisons test. (I–K) Statistical analyses were conducted using two‐factor analysis of variance (ANOVA) with Tukey's multiple comparisons test. *p < 0.05, **p < 0.01, ***p < 0.001 and ****p < 0.0001 and ns, non significant.

Processing and release of IL‐1β requires the autoproteolytic cleavage and activation of caspase‐1 (CASP1), which occurs with NLRP3 inflammasome activation. Therefore, we next examined CASP1 cleavage and release into supernatants comparing NLRP3 inflammasome activation at 16 h PS versus 28 h PS. We observed increased cleavage of CASP1 p20 following NLRP3 inflammasome activation at 28 h PS versus 16 h PS (Figure 3A,B). We next examined whether time‐of‐day CASP1 cleavage also extends to GSDMD activation and pyroptosis. Similarly, to CASP1, we observed increased GSDMD p30 cleavage and release following NLRP3 inflammasome activation at 28 h PS compared to 16 h PS (Figure 3C,D). CASP1 activation was observed to be dependent on Δψm, such that mitochondrial uncoupling with FCCP, prior to inflammasome activation with ATP, reduced CASP1 p20 cleavage at both 16 h and 28 h PS (Figure 3E,F). This was also observed for GSDMD, with FCCP ablating GSDMD p30 cleavage following NLRP3 inflammasome activation at 16 h and 28 h PS (Figure 3G,H). Furthermore, we found that mitochondrial disruption with FCCP significantly reduced NLRP3 inflammasome‐mediated IL‐1β (Figure 3I) and LDH release (Figure 3J) at both 16 h and 28 h PS. Of note, treatment with FCCP at 28 h reduced the heightened levels of IL‐1β and LDH down to the levels observed with FCCP treatment at 16 h PS. LPS‐induced TNFα release was not affected by FCCP treatment or time of day comparing 16 h versus 28 h PS (Figure 3K). Overall, this data demonstrates that NLRP3 inflammasome activation and pyroptosis are dependent on time of day, and that disrupting mitochondrial membrane potential with FCCP ablates time‐of‐day IL‐1β release and pyroptosis.

### 3.5 NLRP3 inflammasome activation and pyroptosis are enhanced with *Bmal1* deletion

**FIGURE 4:**
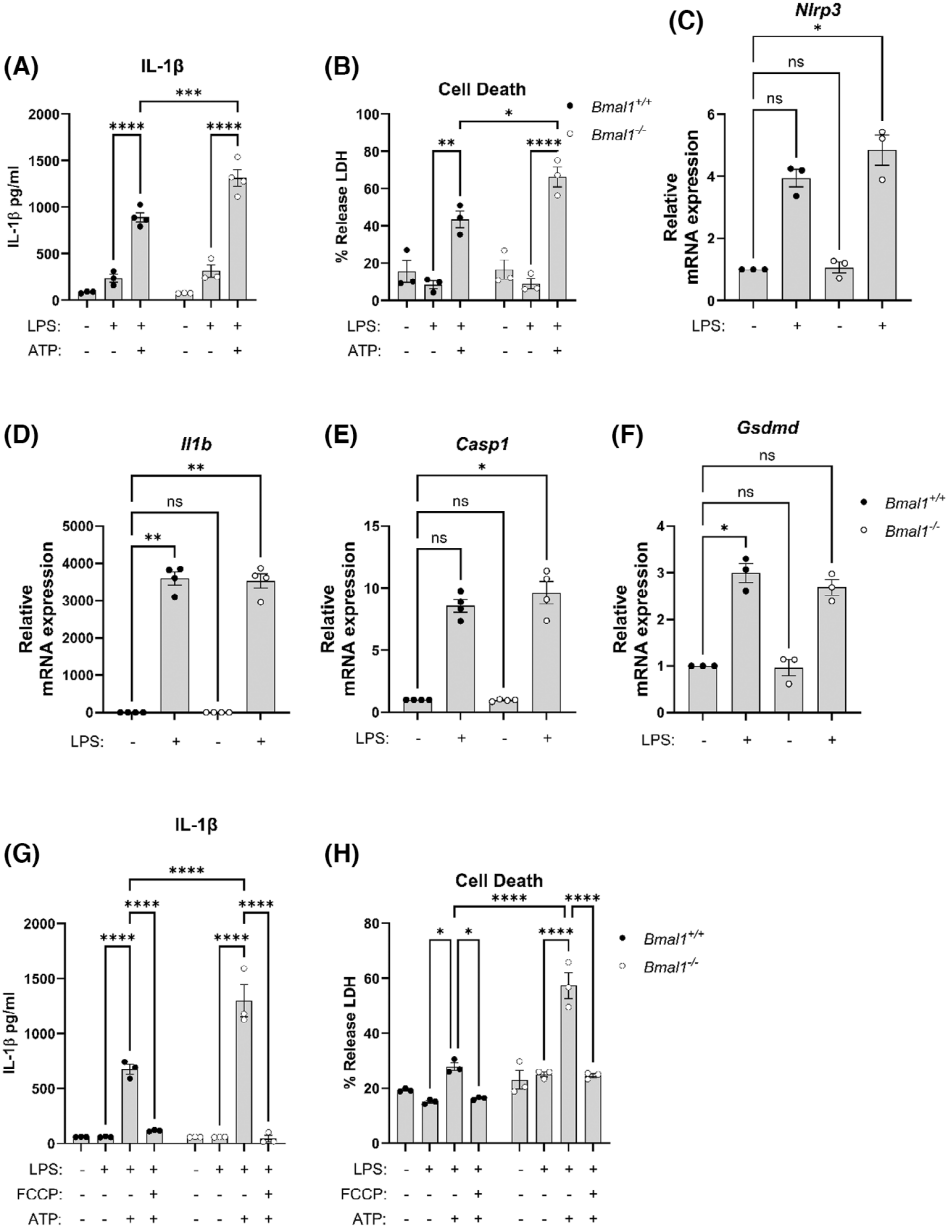
NLRP3 inflammasome activation is enhanced with *Bmal1* deletion and dependent on mitochondrial membrane potential. (A) IL‐1β protein released from NLRP3 inflammasome activated unsynchronized *Bmal1* ^*+/+*^ and *Bmal1* ^*−/−*^ BMDMs (*n* = 3/4). (B) Cell death assayed via release of lactate dehydrogenase from NLRP3 inflammasome activated unsynchronized *Bmal1* ^*+/+*^ and *Bmal1* ^*−/−*^ BMDMs (*n* = 3). (C–F) *Nlrp3, Il1b, Casp1 and Gsdmd* mRNA expression of control and LPS (100 ng/mL, 3 h) treated *Bmal1* ^*+/+*^ and *Bmal1* ^−/−^ BMDMs (*n* = 3/4). (G) IL‐1β release from NLRP3 inflammasome activated *Bmal1* ^*+/+*^ and *Bmal1* ^*−/−*^ BMDMs with FCCP inhibition (*n* = 3). (H) Cell death assayed via release of LDH from NLRP3 inflammasome activated *Bmal1* ^*+/+*^ and *Bmal1* ^*−/−*^ BMDMs with FCCP inhibition (*n* = 3). Data are expressed as mean ± SEM. *N* numbers represent biological samples. (A, B, G, H) Statistical analysis carried out by two‐factor ANOVA with Tukey's multiple comparisons test. (C–F) Statistical analyses were conducted using Kruskal–Wallis with Dunn's multiple comparisons test. *p < 0.05, **p < 0.01, ***p < 0.001 and ****p < 0.0001 and ns = non significant.

We next assessed NLRP3 inflammasome activation with *Bmal1*‐deletion in peritoneal exudate cells (PECs) isolated in the morning and in unsynchronized BMDMs. We observed increased IL‐1β and LDH release in *Bmal1* ^*−/−*^ PECs compared to *Bmal1* ^*+/+*^ controls (Figure S4A,B). To determine the effect of *Bmal1*‐deletion on mitochondria, we examined mitochondrial membrane potential from PECs of *Bmal1* ^*+/+*^ versus *Bmal1* ^*−/−*^ mice (data not shown). We observed no difference in Δψm between *Bmal1* ^*+/+*^ versus *Bmal1* ^*−/−*^ PECs. We confirmed in unsynchronized *Bmal1* ^*−/−*^ BMDMs an increased release of IL‐1β and LDH following NLRP3 inflammasome activation compared to *Bmal1* ^*+/+*^ BMDMs (Figure 4A,B). We next examined the abundance of cellular ROS between *Bmal1* ^*+/+*^ and *Bmal1* ^*−/−*^ BMDMs in response to LPS and FCCP yet observed no significant differences in cellular ROS abundance (Figure S3). We next assessed potential differences in the mRNA expression of inflammasome components between *Bmal1* ^*−/−*^ and *Bmal1* ^*+/+*^ BMDMs. We noticed an increase in the expression of *Nlrp3* (Figure 4C) and *Casp1* (Figure 4E) with *Bmal1* deletion, however, noted no differences in *Il1b* (Figure 4D) or *Gsdmd* (Figure 4F). We next investigated the effect of FCCP with *Bmal1*‐deletion and observed a significant reduction of the heightened levels of NLRP3 inflammasome‐mediated release of IL‐1β (Figure 4G) and LDH (Figure 4H) to levels observed with FCCP treatment of *Bmal1* ^*+/+*^ controls. Collectively, these findings demonstrate that the NLRP3 inflammasome response is dependent on mitochondria membrane potential and that uncoupling membrane potential with FCCP ablates the heightened NLRP3 inflammasome activation and pyroptosis observed with *Bmal1‐*deletion.

## 4 DISCUSSION

The key findings of this study are that the NLRP3 inflammasome is under time‐of‐day control by the molecular clock at both inflammasome “priming” and “activation” stages. Circadian clock genes mediate this control, such that deletion of *Bmal1* enhances NLRP3 inflammasome activity. We show that the NLRP3 inflammasome is dependent on mitochondrial membrane potential (Δψm), as pharmacological disruption of Δψm ablates time‐of‐day caspase‐1 (CASP1) and gasdermin D (GSDMD) activation. This subsequently reduced the time‐of‐day release of IL‐1β and cell death. On the other hand, time‐of‐day differences in pro‐IL‐1β expression were not affected by disruption of Δψm following LPS priming. Therefore, we have uncovered a novel mechanism of time‐of‐day control of the NLRP3 inflammasome, mediated via regulation of Δψm.

We have previously observed in bone marrow‐derived macrophages and dendritic cells that mitochondria are rhythmically controlled when the molecular clock is in a synchronized state.^34^, ^38^ Others have similarly observed rhythms in mitochondrial membrane potential in synchronized peritoneal macrophages.^35^ Mitochondrial ATP synthesis by clockwise rotation of ATP synthase occurs at the expense of Δψm, whereas counterclockwise rotation of ATP synthase and hydrolysis of ATP support Δψm.^40^ Mitochondria likely maintain a balance in Δψm at the cost of ATP to coordinate oxidative and reductive stress, facilitate protein transport and colocalization, regulate second messenger signaling, that is, ROS, Fe^2+^, and Ca^2+^, manage energy consumption, amongst other functions.^40^ Further, many biological processes affected by Δψm are time‐of‐day regulated in macrophages, for example, Ca^2+^ and ROS signaling.^2^, ^35^, ^41^ Time‐of‐day differences in Δψm likely have wide‐ranging implications for inflammatory processes. Indeed, we have recently shown that circadian rhythms in dendritic cell mitochondrial morphology, Ca^2+^ storage, and Δψm, which impacts antigen processing.^38^

The importance of molecular clock genes and circadian rhythms in mitochondrial metabolism has been observed across different tissues and cell types, for example, liver^42^, ^43^, ^44^ and skeletal muscle.^45^, ^46^ However, in recent years, the role of mitochondrial metabolism for circadian function in myeloid cells has been under particular focus.^34^, ^35^, ^36^, ^37^, ^38^, ^47^ We observed significant differences basally in macrophage Δψm from PECs isolated at CT12 compared to CT0, supporting previous observations of circadian regulation of mitochondrial function in macrophages.^34^, ^35^, ^36^, ^37^, ^47^ Time‐of‐day differences in Δψm are low amplitude, which suggests this mechanism of regulation fine‐tunes the NLRP3 inflammasome across time of day. We did not identify significant differences in Δψm of peritoneal‐isolated B cells. This may agree with data showing that the molecular clock of B cells is dispensable for their differentiation and function.^48^ Although we observe elevated Δψm and NLRP3 inflammasome activation, we did not observe these trends in Δψm comparing *Bmal1* ^*+/+*^ and *Bmal1* ^*−/−*^ PECs. The role of *Bmal1* in time‐of‐day mitochondrial function remains complex. Indeed, we observe that *Bmal1* deletion impacts other clock genes such as *Nr1d1, Cry1*, and *Cry2* in time‐of‐day studies. Therefore, *Bmal1* may act upstream of other circadian regulators that can influence inflammasome activation and mitochondrial function, which we cannot distinguish from our study.

We sought to understand the mechanism through which time‐of‐day control of the electron transport chain (ETC) affected NLRP3 inflammasome activation. The ETC is critical for NLRP3 inflammasome activation.^30^ Mitochondrial inhibitors that disrupt Δψm have previously been used to abolish circadian rhythms in macrophage phagocytosis.^34^ However, disrupting Δψm may affect NLRP3 inflammasome activation through a variety of mechanisms. Uncoupling Δψm with protonophores may lead to reduced hydrogen peroxide (H_2_O_2_) production and increased mitochondrial respiration.^49^ Reactive oxygen species (ROS) have a historically complex role in regulating NLRP3 inflammasome activation.^20^ The leading theory proposes that ROS can regulate but are not essential for NLRP3 inflammasome activation, depending on the context of activation. We have previously observed a role for *Bmal1* in regulating ROS production localized to mitochondria in response to 24 h LPS treatments.^41^ However, with 3 h LPS treatment and 1 h FCCP inhibition, we found no significant time‐of‐day difference in cellular ROS abundance with *Bmal1* deletion. Duration of LPS treatment and differing methods of ROS assessment are likely to account for these different outcomes. Therefore, the time‐of‐day regulation of NLRP3 inflammasome activation we observe is unlikely due to changes in total ROS. Circadian rhythms in Δψm affect ATP abundance in synchronized macrophages and dendritic cells.^34^ Moreover, NLRP3 oligomerization is dependent on the ATP‐ase activity of its NACHT domain.^50^, ^51^ Therefore, we considered that changes in ATP abundance generated by rhythms in mitochondria might contribute to time‐of‐day NLRP3 inflammasome activation. However, we observed no significant differences in cellular ATP concentration basally or with LPS stimulation between 16 h and 28 h PS. Since our investigations broadly targeted total cellular ROS and ATP, we cannot rule out that localized differences in ROS and ATP may contribute to time‐of‐day control of the NLRP3 inflammasome. It is also possible that time‐of‐day differences in ROS and ATP exist at other times post‐synchronization, which we did not investigate in this study. However, extensive investigations into different circadian timing, and the precise location of the NLRP3 inflammasome would be required to elucidate these issues, which remain a complex challenge.^18^

Circadian control of PRR signaling is well established in macrophages, with rhythmic expression observed for TLRs and downstream regulators, that is, *Iκbα*.^52^, ^53^, ^54^, ^55^, ^56^ Molecular clock control of the NLRP3 inflammasome was first demonstrated with genetic deletion of the core circadian genes, *Bmal1* ^32^ and *Nr1d1*.^31^, ^57^ In these studies, circadian timing of NLRP3 inflammasome activity was largely attributed to transcriptional repression of *Nlrp3* and *Il1b* by BMAL1 and REV‐ERBα.^31^, ^32^ We have previously identified a transcriptional role of BMAL1 in regulating pro‐IL‐1β production via the NRF2‐NF‐κB axis^41^ and further found that BMAL1 regulates the PKM2‐STAT3 axis via glycolysis to limit pro‐IL‐1β expression.^37^ To examine whether BMAL1 regulates the NLRP3 inflammasome independent of these known transcriptional controls, we opted for short (3 h) LPS treatments where the transcriptional effects mediated by *Bmal1* are less apparent. Although we observed significant differences in *Nlrp3* mRNA expression between LPS treatments at 16 h and 28 h PS, we did not observe corresponding differences at the protein level. This finding strengthens previous research that transcriptional and translational regulation mediated by the molecular clock is uncoupled in macrophages.^34^ Indeed, post‐translational modifications of NLRP3 inflammasome components regulate inflammasome activity.^25^, ^28^, ^58^, ^59^, ^60^, ^61^ Although beyond the scope of this study, future investigations into time‐of‐day regulation of NLRP3 inflammasome activation in macrophages focusing on the different post‐translational modifications could yield novel insights into this mode of time‐of‐day regulation.

Pyroptosis is distinguishable from other forms of non‐inflammatory cell death via the release of alarmins and cytoplasmic complexes, such as lactate dehydrogenase (LDH).^62^ We sought to understand whether time‐of‐day IL‐1β release was due to time‐of‐day regulation of pyroptosis, as prior studies had not robustly investigated pyroptosis. Indeed, we observe greater LDH release and increased cleavage of the active N‐terminal (p30) GSDMD fragment, at 28 h PS compared to 16 h PS. This suggests that pyroptosis induced by the NLRP3 inflammasome is also under clock control. To investigate how the molecular clock regulates NLRP3 inflammasome activation via mitochondrial control, we opted to treat cells with LPS prior to mitochondrial disruption with FCCP as mitochondria play an essential role in PRR signaling.^63^ This allows normal mitochondrial function during PRR signaling and facilitates investigations into the role of mitochondria in the activation stage of the NLRP3 inflammasome. Our observation that FCCP did not affect TNFα release suggests that Δψm is specifically important in NLRP3 inflammasome activation. Furthermore, in this model we found no effect of FCCP in LPS priming of pro‐IL‐1β and NLRP3, whereas CASP1 p20 and GSDMD p30 cleavage were reduced with FCCP. These results show that disrupting rhythmic changes in Δψm affects time‐of‐day pyroptosis and IL‐1β release following NLRP3 inflammasome activation.

We observe increased release of IL‐1β following NLRP3 inflammasome activation at CT12 compared to CT0, which agrees with previous studies that show circadian differences in IL‐1β release.^31^, ^41^, ^47^ However, there are key differences in experimental design that may affect timing between our experiments and those of previous studies. We examined NLRP3 inflammasome activation ex vivo in response to LPS and ATP. Nguyen et al. examined circadian ex vivo IL‐1β release in serum following infection with Listeria monocytogenes, whereas Pourcet et al. examined circadian IL‐1β release ex vivo from unstimulated peritoneal exudate cells.^31^, ^47^ These differences in experimental set up likely account for the observed changes in timing between studies. In this study, we did not investigate whether time‐of‐day regulation of other inflammasome sensors, such as AIM2, also occurs. As other inflammasomes are also sensitive to mitochondrial control,^64^, ^65^ we anticipate that time‐of‐day regulation may extend to other inflammasomes, potentially through effects on mitochondria.

In summary, our study identifies a novel model of time‐of‐day regulation of the NLRP3 inflammasome and pyroptosis, which is mediated by mitochondrial membrane potential. Our data strengthen previous findings that the molecular clock exerts transcriptional control over the NLRP3 inflammasome pathway.^31^, ^32^, ^33^, ^66^ However, where Shim et al. observed no significant difference in canonical NLRP3 inflammasome activation with *Bmal1* deletion, we observe enhanced canonical NLRP3 inflammasome activity. Differences in treatment concentrations and strategy may account for these different outcomes.

We note that time‐of‐day regulation of inflammation may be amplified by molecular clock timing between cells, for example, enhanced release of IL‐1β by the NLRP3 inflammasome at CT12 found in this study may complement our prior observations that lung fibroblasts produce significantly more CXCL5 in response to IL‐1β at CT12.^16^ Taken together, these studies suggest amplified time‐of‐day regulation of the IL‐1β inflammatory response across cell types and tissues. There is growing evidence that the NLRP3 inflammasome plays a pathogenic role in chronic inflammatory diseases that exhibit circadian rhythmicity, such as rheumatoid arthritis, hepatitis, and asthma.^33^ Our findings provide mechanistic insight as to how the molecular clock, and particularly *Bmal1*, regulates the activity of the NLRP3 inflammasome. This may be critically important in understanding diseases where both inflammasomes and circadian rhythms play central roles and may provide new chronotherapeutic opportunities for administration of inflammasome inhibitors.

## AUTHOR CONTRIBUTIONS

James R. O'Siorain designed and performed experiments, analyzed data, and co‐wrote the manuscript; Shannon L. Cox performed experiments, analyzed data, and critically appraised the manuscript; Frances K. Nally and Jennifer K. Dowling performed in vitro experiments and analyzed data; Yan He assisted with ex vivo experiments; Tabea T. Drewinksi performed in vitro experiments and analyzed data; Oran D. Kennedy and Mark Mellett appraised the manuscript; James O. Early performed experiments, conceived and supervised the study, Annie M. Curtis conceived and supervised the study, and co‐wrote the manuscript.

## DISCLOSURES

The authors have stated explicitly that there are no conflicts of interest in connection with this article.

## Supporting information


Figures S1‐S4.

Dataset S1.

Dataset S2.

## Data Availability

The data supporting the conclusions of this article will be made available by the authors, without undue reservation.
