# Supplementary material for: Time‐of‐day control of mitochondria regulates NLRP3 inflammasome activation in macrophages
Source: FASEB J. 2024 Dec 17;38(24):e70235. doi: 10.1096/fj.202400508RR (PMC11669068; doi:10.1096/fj.202400508RR)
Supplement: Supplementary file 1 — Figures S1‐S4. [file FSB2-38-e70235-s003.docx]

**
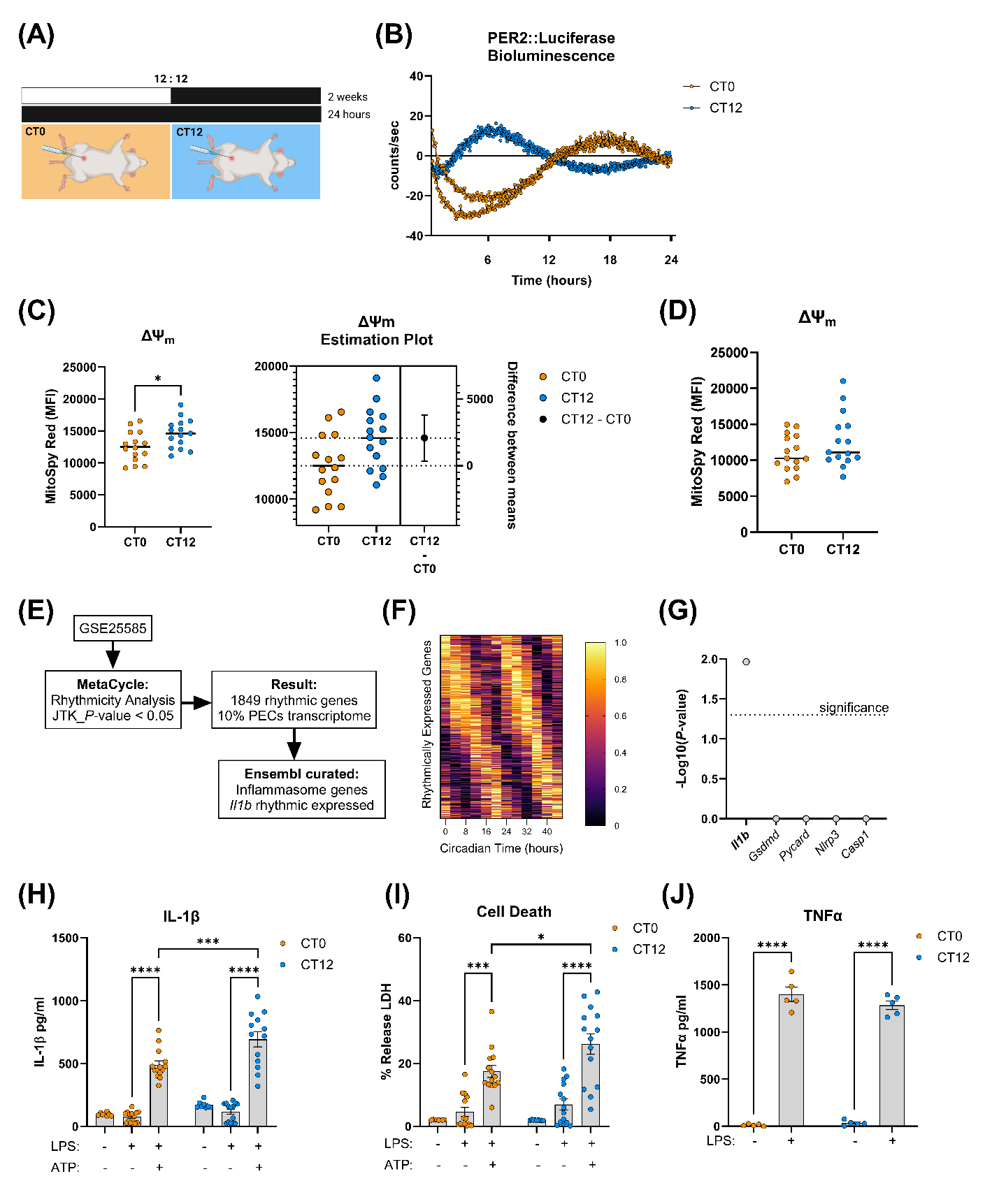
**

**Supplementary Figure 1.**

**(A)** Schematic illustrating ex vivo PECs experiment design, made with BioRender. **(B)** PER2::luciferase bioluminescent counts of peritoneal exudate cells (PECs) isolated at circadian times (CT) CT0 and CT12, then assessed for 24 hours (n=2). **(C)** Mitochondrial membrane potential (Δψm) of peritoneal macrophages isolated at CT0 and CT12 and measured with MitoSpy Red CMXRos (n=14). **(D)** Peritoneal B cell mitochondrial membrane potential isolated at CT0 versus CT12. **(E)** Bioinformatics workflow analysis of GSE25585 of gene expression data from PECs isolated every 4 hours over 48 hours. **(F)** Heatmap of rhythmically expressed genes identified from GSE25585. **(G)** Identification of *Il1b* as a rhythmically expressed inflammasome gene curated via Ensembl. **(H)** IL-1β protein released from NLRP3 inflammasome activated PECs isolated at CT0 versus CT12 (n=13). **(I)** Cell death assayed via release of lactate dehydrogenase (LDH) from PECs isolated at CT0 versus CT12 (n=14). **(J)** TNFα released from LPS activated PECs isolated at CT0 versus CT12 (n=5). Data are expressed as mean ± SEM. N numbers represent biological samples with technical duplicates. **(B)** Circadian parameters measured with MetaCycle JTK cycle analysis (period = 24 h, P < 0.001). **(C, D)** Statistical analysis was conducted using Welch’s t test. **(H-J)** Statistical analyses were conducted using two-factor analysis of variance (ANOVA) with Tukey’s multiple comparisons test.


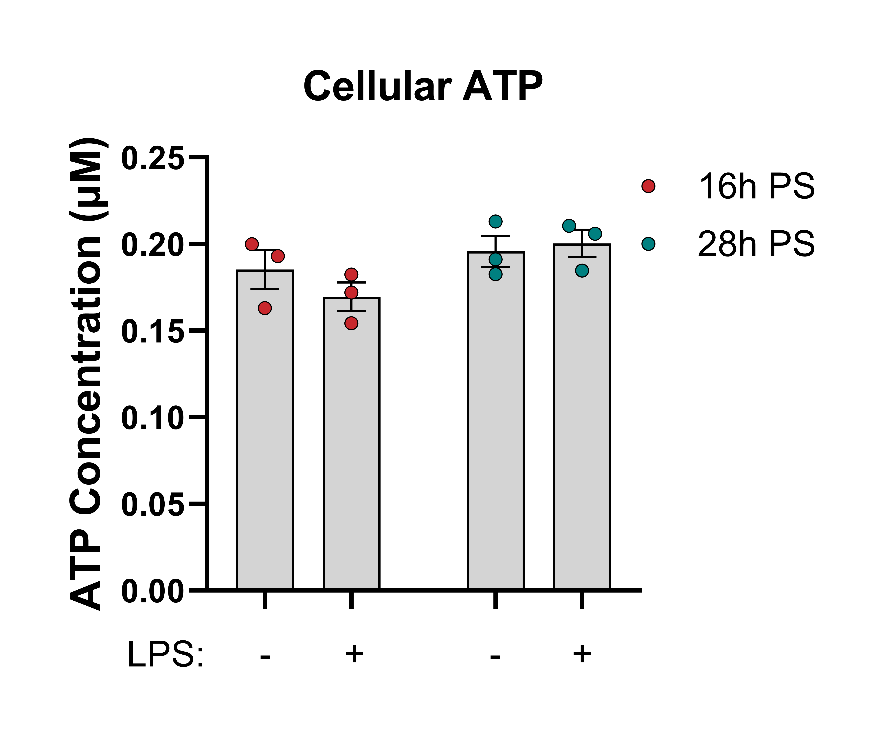


**Supplementary Figure 2.**

Cellular ATP abundance from LPS treated BMDMs at 16h and 28h PS. Data are expressed

as mean ± SEM. N numbers represent biological samples.

**
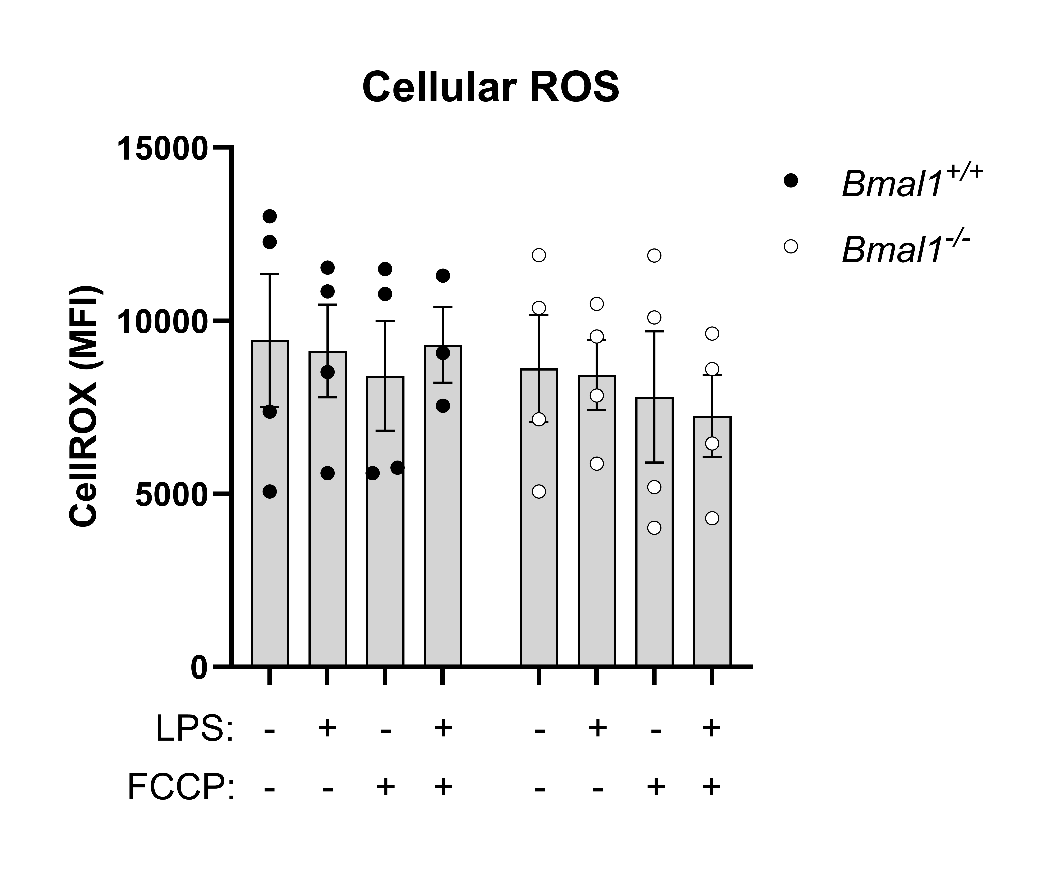
**

**Supplementary Figure 3.**

Cellular ROS abundance from unsynchronized BMDMs isolated from *Bmal1^+/+^* and *Bmal1^-/-^* mice. Data are expressed as mean ± SEM. N numbers represent biological samples.

**
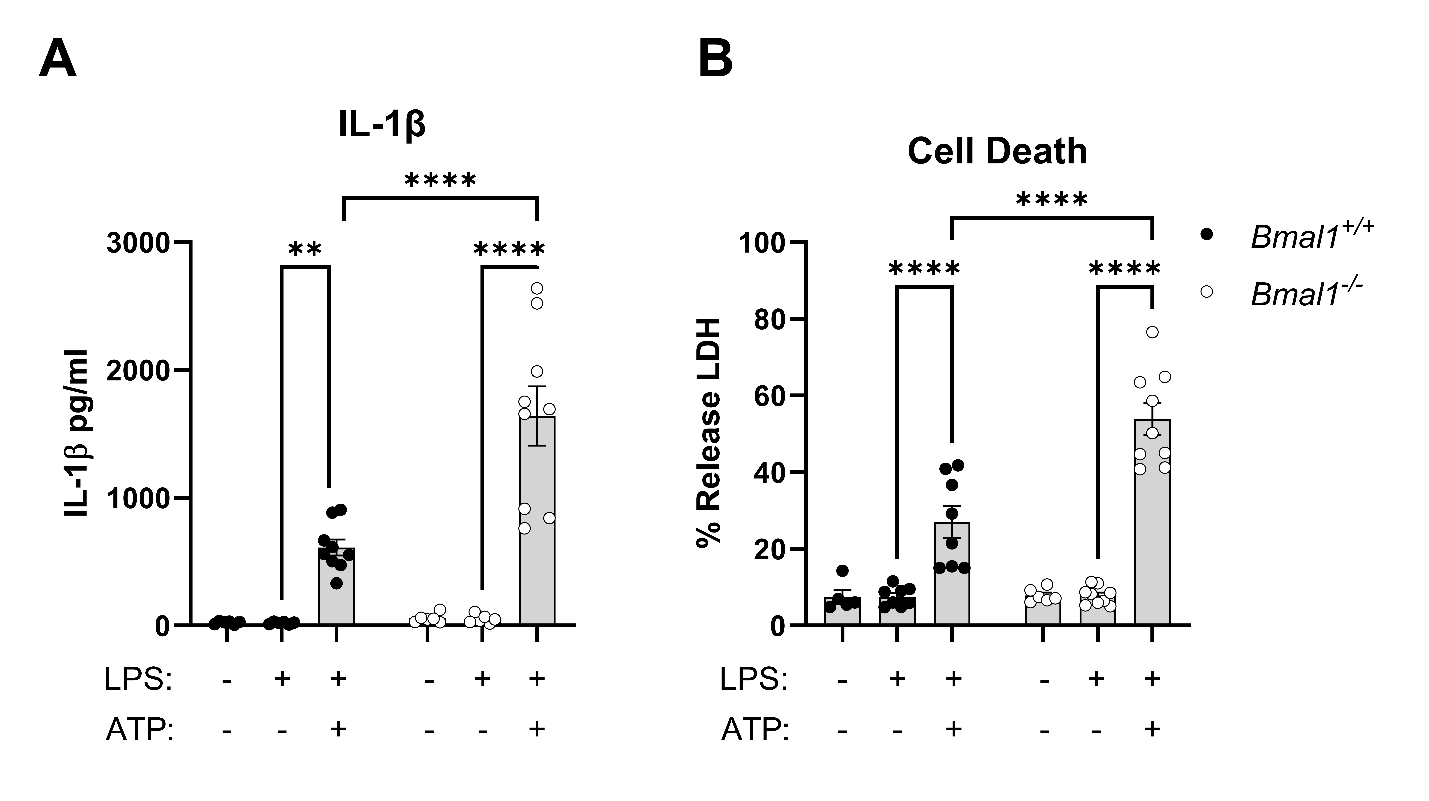
**

**Supplementary Figure 4.**

**(A)** IL-1β protein released from NLRP3 inflammasome activated *Bmal1^+/+^* and *Bmal1^-/-^* PECs isolated in the morning (n=9). **(B)** Cell death assayed via release of lactate dehydrogenase from NLRP3 inflammasome activated PECs from *Bmal1^+/+^* and *Bmal1^-/-^* mice isolated in the morning (n=8). Data are expressed as mean ± SEM. N numbers represent biological samples. Statistical analysis carried out by two-factor ANOVA with Tukey’s multiple comparisons test.
